# Sodium Glucose Cotransporter-2 Inhibitors: Spotlight on Favorable Effects on Clinical Outcomes beyond Diabetes

**DOI:** 10.3390/ijms23052812

**Published:** 2022-03-04

**Authors:** Věra Čertíková Chábová, Oskar Zakiyanov

**Affiliations:** Department of Nephrology, 1st Faculty of Medicine, Charles University and General University Hospital in Prague, U Nemocnice 2, 12800 Prague 2, Czech Republic; oskar.zakiyanov@lf1.cuni.cz

**Keywords:** SGLT2, SGLT2 inhibitors, diabetes, chronic kidney disease, heart failure

## Abstract

Sodium glucose transporter type 2 (SGLT2) molecules are found in proximal tubules of the kidney, and perhaps in the brain or intestine, but rarely in any other tissue. However, their inhibitors, intended to improve diabetes compensation, have many more beneficial effects. They improve kidney and cardiovascular outcomes and decrease mortality. These benefits are not limited to diabetics but were also found in non-diabetic individuals. The pathophysiological pathways underlying the treatment success have been investigated in both clinical and experimental studies. There have been numerous excellent reviews, but these were mostly restricted to limited aspects of the knowledge. The aim of this review is to summarize the known experimental and clinical evidence of SGLT2 inhibitors’ effects on individual organs (kidney, heart, liver, etc.), as well as the systemic changes that lead to an improvement in clinical outcomes.

## 1. Introduction

Physicians who trained at the end of the last century were used to evaluate the compensation of diabetes by the presence or absence of glucosuria. They may consider improving compensation by increasing glucose excretion in urine to be considered very controversial. However, sodium glucose transporter type 2 (SGLT2) inhibitors not only cause glucosuria and improve diabetic compensation, but also decrease the risk of chronic kidney disease (CKD) progression [1,2,3], decrease the risk of cardiovascular events [3,4,5,6,7], and even lower all-cause mortality in treated patients [2,4,6,8]. The most puzzling information is that they have these effects in non-diabetic individuals as well [4,9], even if the mortality is not decreased, as was shown in a recent meta-analysis [10]. After years of negative studies with other kinds of drugs, this was a very satisfying result. 

To some extent, this resembles the impact of angiotensin-converting enzyme inhibitors (ACEI). However, it is known that the renin angiotensin aldosterone system (RAAS) or its parts are found throughout the body [11]. It is not difficult to understand that ACEI will have different effects to merely lowering blood pressure. On the other hand, SGLT2 is mainly expressed in the S1 and S2 part of the proximal renal tubule. There were hardly any other sites where substantial amounts of SGLT2 receptors were found [12], apart from the brain [13], and maybe also the intestinal mucosa [14]. SGLT2 is responsible for the renal reabsorption of 90% of filtered glucose. The rest is transported further downstream by sodium glucose transporter type 1 (SGLT1) [15]. However, blockade or knockout of SGLT2 only decreases glucose reabsorption by 30–50%, and not by 90%, as would be expected. This is probably caused by the upregulation of SGLT1. Mice with double knockout of SGLT1 and SGLT2 excreted three times more glucose than SGLT 2 knockouts alone [16]. Dual SGLT1 and SGLT2 inhibitors, canagliflozin and sotagliflozin, were also found to inhibit intestinal SGLT1 [17,18], but this is not a class effect.

How can the inhibition of a single transporter in a tiny part of the nephron in a small organ of the kidney have such a huge impact on the fate of the whole body? There have been numerous recent reviews on this topic, which cannot be cited here in full; however, they mostly deal with only part of the effects. 

Thus, the aim of this review is to summarize the effects to explain the improvement in many renal, cardiovascular and mortality outcomes. However, it is important to bear in mind that not everything is known at present, and most effects are subject to interconnecting regulatory positive and negative feedback. SGLT2 inhibitors induce systemic changes that might affect individual organs, such as kidneys and heart, secondarily, and specific single-organ changes that contribute to the overall improvement. The systemic changes include diabetes compensation, a decrease in body weight, blood pressure lowering and decreased sympathetic tone, as well as the suppression of inflammation and atherosclerosis. Other important metabolic improvements include a lower concentration of uric acid, lower incidence of hyperkalemia, normalization of magnesium concentrations, etc. Blood parameter changes after SGLT2 inhibitors are summarized in Table 1. Improvements in the composition and function of individual organs were found in kidney, heart, liver, and retina [19].

## 2. Diabetes Compensation

SGLT2 inhibitors are primarily antidiabetic drugs. They were properly tested for efficacy and safety before being approved by respective regulatory authorities. Thus, the studies confirming a glucose-lowering effect and diabetes compensation will not be cited in this paragraph; they will be cited when additional effects are documented. 

A better compensation of diabetes undoubtedly improves outcomes for diabetics [31]. Gliflozins decrease glycosylated hemoglobin (GHbA1c) by approximately one percentage point when used as a monotherapy or as an add-on to other drugs. This is comparable to dipeptidyl-peptidase 4 inhibitors, but lower than sulfonylureas or glucagon-like-peptide-1 (GLP-1) agonists. This decrease cannot fully explain the early improvement in heart, kidney and survival outcomes (within 1–2 years of follow-up), which are not proportionate to the degree of compensation [32]. SGLT2 inhibitors not only decrease the amount of glucose in the system, but also improve insulin sensitivity [33]. Gliflozins improve the survival and regeneration of beta-cells [34]; however, this effect has also been described after other drugs [35]. Again, this does not explain the short-term effect. However, in the long term, it alleviates the burden of diabetic complications.

The beneficial effect might be partly attenuated by increased endogenous glucose production [36,37]. Gliflozins also shift the utilization of substrates from carbohydrates to lipids [37], which might improve the nutrition of the cells. In conclusion, SGLT2 inhibitors improve diabetes compensation by decreasing glucose availability, improving insulin sensitivity and energy utilization. 

## 3. Decreasing the Body Weight

The first studies testing gliflozins for effectivity and safety usually also reported loss of body weight [38,39,40,41], even if not all of them [42]. Gliflozins stimulate lipolysis, lipid oxidation and ketogenesis, which helps to reduce the body fat [43]. The decrease in body weight might be partly driven by the change in gut microbiota. This was proven in mice [44], but not in humans [45]. 

Loss of glucose decreases the calories available to the body. This might lead to hyperphagia to compensate, as reported in Reference [18]. However, not every experiment is in concordance with this. Sawada et al. reported no hyperphagia compared to untreated rats when rats were fed on a high-fat diet. In their experiment, the explanation for the slower gains in body weight was the liver–brain–adipose neural axis. Tofogliflozin decreased fat mass in intact mice, but this effect was attenuated by hepatic vagotomy [46]. SGLT2 inhibition by canagliflozin promoted adipose thermogenesis, mitochondrial biogenesis, and lipolysis via the β-adrenoceptor-cyclic adenosine3′5′-monophosphate-protein kinase A pathway [47]. Moreover, SGLT 2 inhibitors induce white adipose tissue lipolysis. This effect is not highly desirable as it might trigger diabetic ketoacidosis [48], but it can prevent fat accumulation driven by insulin. 

As both American and European guidelines recommend treatment of obesity in individuals with type 2 diabetes (T2D) [49], lowering body weight might be another pathway to better outcomes in treated patients.

## 4. Blood Pressure Lowering

SGLT2 inhibitors decrease blood pressure, as was found in earlier studies [40,41] and pooled data [50], and confirmed in recent metanalyses [51,52]. However, the real decrease is only several mmHg compared with usual care.

Canagliflozin induced natriuresis, but not urine output, so no osmotic diuresis was seen [53]. However, in acute cardiac failure, osmotic diuresis was found, but fractional sodium excretion was not increased [54].

Dapagliflozin was found to decrease estimated plasma volume by approximately 10%, which might help to decrease blood pressure [55]. Extracellular and plasma volume were also decreased after empagliflozin [56]. The skin sodium content was decreased after 6 weeks of dapagliflozin [57].

A lower plasma volume and sodium body content are probably the primary mechanisms that decrease blood pressure. In animals, night dipping might also be restored by gliflozins; in humans, the pattern was maintained [58]. This effect is increased in combination with RAAS inhibitors. A benefit was also found in combination with beta-blockers or calcium-channel blockers [59] but was not potentiated by thiazide diuretics or furosemide, probably because gliflozins trigger a substantial increase in plasma renin and aldosterone [29]. However, not every study found a correlation with other antihypertensives [60].

Decreasing the sympathetic activity driven by the kidney might be one of the major mechanisms that lead to decreased incidence of heart failure, as summarized in a recent review [61]. In our opinion, the kidney protection cannot be explained by the blood pressure effect only, as glomerular and interstitial fibrosis and inflammatory infiltrates were attenuated by empagliflozin, without any effect on blood pressure in angiotensin-II-dependent hypertension in rats [62]. 

Based on the available evidence, clinical guidelines recommend treatment of hypertension and maintenance of normal blood pressure in both diabetic and non-diabetic population to prevent cardiovascular and kidney complications [63,64,65,66]. The contribution of SGLT2 inhibitors to blood-pressure lowering might be another benefit leading to better outcomes.

## 5. Vascular Effects and Inflammation

Cardiovascular outcomes, as well as the kidney outcomes mentioned above, might be driven by vascular, inflammatory and subcellular changes. Indeed, inflammation, angiogenesis, atherosclerosis and arterial stiffness were influenced by this medication.

Activation of the NLR family, pyrin-domain-containing 3 (NLRP3) inflammasome and subsequent interleukin (IL)-1β release induces atherosclerosis and heart failure [67]. Patients with T2D and high cardiovascular risk received SGLT2 inhibitor empagliflozin or sulfonylurea for 30 days, with NLRP3 inflammasome activation analyzed in macrophages. While the SGLT2 inhibitor’s glucose-lowering capacity is comparable to sulfonylurea, it showed a greater reduction in IL-1β secretion compared to sulfonylurea, accompanied by increased serum β-hydroxybutyrate and decreased serum insulin [67].

Canagliflozin decreased leptin and increased adiponectin and decreased proinflammatory IL-6. Some increase in tumor necrosis factor (TNF) alpha was also found in this study; however, the cause and significance of this is unknown [68].

Canagliflozin decreased angiogenesis in diabetic mice [69]. However, this is a double-edged sword. It might decrease the repair after ischemia, as in the cited study. The CANVAS trial reported a higher incidence of amputations in humans as well, even if this was not confirmed in other studies. On the other hand, the inhibition of angiogenesis might lead to a slower progression of diabetic retinopathy [70]. The second study used the same model of diabetic mice but a different drug, tofogliflozin. Both drugs decreased the vascular endothelial growth factor. A pilot trial in humans also suggests beneficial effects in retinopathy [71].

Other common pathway leading to better outcomes might be the improved mitochondrial function [72,73]. Improved energy production and mitochondrial biogenesis were found in experimental studies [74].

No long-term trials with atherosclerosis development as an outcome are available in humans at present. Obviously, atherosclerosis risk factors, such as glucose metabolism, uric acid concentrations, blood pressure normalization and body-weight lowering are all influenced by gliflozins. The lipid profile changes have also been investigated in more detail, as well as parameters of vascular stiffness and endothelial function.

Dapagliflozin was proven to decrease atherosclerosis development and endothelial disfunction in apo-E deficient diabetic mice [75]. In a different model, empagliflozin accelerated the regression of atherosclerosis [76]. Lowered triglycerides content and an increase in HDL cholesterol were also found in hypertriglyceridemic mice [77].

A recent metanalysis found that SGLT2 inhibitors increase total cholesterol level, high-density lipoprotein (HDL)-cholesterol and low-density lipoprotein (LDL)-cholesterol. Gliflozins also decreased triglyceride concentrations, which is consistent with animal studies [28].

Increased LDL-cholesterol is not a desirable effect. However, a study with dapagliflozin found an increase in the less atherogenic, large buoyant LDL-cholesterol and suppression of atherogenic, small dense LDL-cholesterol [27], which suggests a more favorable profile.

Vascular parameters were also studied in humans. Arterial stiffness is an established risk factor for cardiovascular risk. In a post hoc analysis of a phase III study with empagliflozin, ambulatory arterial stiffness index exhibited a decreasing trend, and pulse pressure and double product (systolic BP * heart rate) were significantly lower [50]. In a pooled analysis of five randomized studies with canagliflozin, the pulse pressure and double product were also decreased [78]. However, double product is not a very helpful predictor of cardiovascular outcomes [79]. Acute dapagliflozin medication decreased brachial artery-endothelium-dependent and independent vasodilatation and pulse wave velocity [80]. Flow-mediated dilation was also improved in another study [81]. 

Improved vascular health and less inflammation might be another benefit of SGLT2 inhibitors. However, long-term human studies are still lacking.

## 6. Other Metabolic and Blood Composition Consequences

As mentioned previously, gliflozins also have significant effects on parameters that are not directly related to their main antidiabetic role [82,83]. Some of them can add to the beneficial effects of gliflozins. Others can mediate the adverse events associated with this medication.

Glycosuria increases the excretion of uric acid [84]. This is most probably not mediated by SGLT2, because SGLT2 does not transport fructose and fructosuria has the same uricosuric effect in mice [85]. The connecting transporter can be glucose transporter type 9 (GLUT9), which transports both hexoses and uric acid in the opposite direction. If there is abundant glucose in the lumen, uric acid excretion increases in both the proximal tubule and the collecting duct, where different isoforms of GLUT 9 are located [86]. However, in a recent study in mice, urate transporter URAT1, rather than GLUT9, was necessary for the uricosuric effect of canagliflozin [87]. Baseline uric acid levels were associated with worse outcomes in a sub-analysis of the EMPA-Reg study and empagliflozin improved these outcomes [88]. In pooled data from four studies with canagliflozin, a 13% reduction in uric acid concentration was found [25]. A recent network meta-analysis [26] confirmed these findings. Uricosuria is a class effect; however, individual compounds were not found equal in this study. A Japanese analysis of three studies with luseogliflozin found both decreased and increased uric acid after 12 weeks, and this effect was dependent on the baseline uric acid concentration, glycosylated hemoglobin and glomerular filtration [89]. Thus, more research elucidating these differences is necessary.

Magnesium deficiency increases cardiovascular risks and diabetics frequently have hypomagnesemia [90]. Gliflozin treatment increases plasma magnesium [22]. In pooled data from 10 studies, a correction of hypomagnesemia by dapagliflozin was found [23].

Hyperkalemia is frequent in patients with decreased glomerular filtration and diabetes. Drugs that inhibit the renin–angiotensin–aldosterone system substantially increase this risk. On the other hand, hypokalemia is also a risk, mainly in heart failure patients. An increased distal nephron flow increases potassium losses. Post-hoc analysis of the CREDENCE trial showed lower incidence of hyperkalemia or initiation of potassium binders [20]. According to a review by Fillipatos et al., a very small increase in potassium concentration was reported in some studies, but no significant change was found [21].

SGLT2 inhibitors increase hematocrit, and this effect is slightly dependent on dose [30]. It can be partly attributed to decrease in plasma volume, as mentioned previously. However, in an analysis of the EMPA-REG study, a transient increase in reticulocytes was also observed, suggesting increased erythrocyte production [91]. Increased erythrocyte production was also found in another study with empagliflozin: transferrin was elevated, while ferritin, total iron and transferrin saturation decreased. There was some trend toward increased erythropoietin [92].

The evidence of bone metabolism changes is somewhat conflicting. Some small studies did not find any changes in the parameters of bone formation or resorption [39,93]. On the other hand, in healthy volunteers, canagliflozin increased phosphate, fibroblast growth factor 23 (FGF-23), and parathormone (PTH), and decreased 1,25-OH vitamin D, in a crossover study, and very similar findings have been demonstrated after dapagliflozin treatment [22,24]. In a post hoc analysis of the IMPROVE trial, dapagliflozin increased serum phosphate, PTH and FGF- 23 and tended to decrease 1,25-OH vitamin D, without any correlation with the changes in estimated glomerular filtration rate (eGFR) and albuminuria [24].

Some changes in laboratory parameters have been associated with better outcomes and some with increased risk of complications, as mentioned above. However, as they might be mixed in individual patients, it is difficult to predict the outcome on this basis only.

## 7. Kidney Protection

As SGLT 2 is found in the kidney, the first positive organ effect should be found there. In fact, hemodynamic, glomerular and tubulointerstitial mechanisms work in concert and all of them contribute to the beneficial effect, demonstrated as a slower deterioration in glomerular filtration [94], and better renal survival in clinical studies, together with decreased albuminuria [3,95]. Patients without diabetes benefited to the same extent, as did patients with decreased renal function [96]. This effect was not attenuated by significantly lower baseline glomerular filtration and the benefits for patients with CKD grade 4 were consistent with other patients in DAPA-CKD [97]. A post hoc analysis of EMPA-REG outcome found a consistent decrease in renal outcome across KDIGO risk categories [98]. Non-diabetic patients with heart failure also exhibited fewer renal outcomes over 16 months of treatment [9].

Glomerular protection is the main pathway to better outcomes. Lower systemic blood pressure and a decrease in hyperfiltration have already been mentioned. However, a drop in glomerular filtration rate appears at the beginning of treatment, which is probably absent in individuals with normal glomerular filtration rate [99] and is not sustained. In moderate renal impairment, there was a decrease in glomerular filtration at the beginning of treatment, but this was found to be reversible after drug withdrawal following 24 weeks of treatment [100]. In the DECLARE-TIMI study, the decrease was larger after one year, the same after 2 years, and lower than in controls after 3 or 4 years [3], showing better renal function preservation after dapagliflozin treatment. Similar findings were found with canagliflozin in the CREDENCE study. This effect was maintained in lower-GFR groups [101,102].

The decrease in albumin/creatinine ratio in those starting with albuminuria is remarkable and, unlike the decrease in glomerular filtration, it is usually sustained [96,103]. Proteinuria/albuminuria is a surrogate marker, and its decrease cannot replace hard outcomes. However, in the CREDENCE trial, the decrease in albuminuria caused by canagliflozin was independently associated with lower risk of the primary kidney outcome, major cardiovascular events and hospitalization for heart failure or cardiovascular death. Residual albuminuria after 26 weeks was an independent risk factor for kidney and cardiovascular events [101]. In non-diabetics, the lowering of albuminuria is reduced compared to in diabetics, but the effect on outcomes is comparable [104].

The decreased absorption of sodium in proximal tubule increases sodium delivery to macula densa. The tubuloglomerular feedback then increases afferent arteriolar vascular tone and decreases glomerular filtration, abolishing the hyperfiltration that leads to albuminuria and glomerular damage, which was also found in diabetes type 1 (T1D) patients [105]. However, in type 2 diabetics, postglomerular rather than preglomerular vasodilatation was found [106].

Tubular cells are also protected under SGLT2 inhibition. Gilbert, in his letter to the editor [107], suggested the underlying mechanism of this: sodium and glucose reabsorption in proximal tubule is energy- and oxygen-consuming. If this transport is blocked, the decreased workload and oxygen demand decrease tubulointerstitial damage. However, in a human study, no difference was found in cortical or medullary oxygenation. Proximal sodium reabsorption decreased but was restored after 1 month due to the rise in renin and aldosterone [29].

In the experiment, podocyte protection by the restoration of autophagy was found after medication with empagliflozin [108]. Autophagy is a cellular recycling process involving self-degradation and the reconstruction of damaged organelles and proteins. This process is vital for podocytes [109]. Dapagliflozin decreased mesangial expansion, tubulointerstitial fibrosis and renal collagen, and fibronectin accumulation. It also modulates tubular cells’ response to hypoxia in streptozotocin-induced diabetes in rats [110].

SGLT2 inhibition decreases the O-linked N-acetylglucosamine-acylation of megalin, leading to accelerated internalization. This ameliorated protein overload of proximal tubular cells, mitochondrial morphological abnormality, renal oxidative stress and tubulointerstitial fibrosis [111]. In tubules, SGLT2 inhibition reduced apoptosis and lipid droplet deposition in tubular cells [112].

There are additional tissue and cellular effects in the kidneys. Li et al. have demonstrated the decreased availability of mitochondrial deacetylase sirtuin 3 in the proximal tubule cells of diabetic mice [113]. This leads to aberrant glucose metabolism and increased endothelial–mesenchymal transition in adjacent vessels, which increases the amount of interstitial fibrosis. Empagliflozin, but not insulin, was able to restore these changes, and, at the same time, improved glomerular damage.

In summary, SGLT2 treatment improves kidney functional and structural parameters by multiple mechanisms, which was confirmed in both experiments and humans. Functional changes, mainly the decrease in hyperfiltration and tubular protein overload, occur rapidly and might be the underlying cause of the short-term outcomes. Structural improvements will hopefully show long-term effects in humans in future.

## 8. Heart and Cardiovascular Protection

Gliflozin treatment reduced the risk of heart failure in diabetic patients, as demonstrated in more clinical trials and confirmed in metanalysis [114]. Patients with established heart failure with reduced ejection fraction also profited from the treatment, as was shown in numerous studies: EMPA-HEART in diabetics [115], Define-HF in the diabetic and non-diabetic population [116], or DAPA-HF [4,117]. The findings were consistent in a large study on real-life patients in registries [6]. The DAPA-HF results show that favorable results are maintained in older age categories [118]. Empagliflozin not only decreased risk of worsening but was associated with improved cardiorespiratory fitness [119].

There was a significant decrease in left ventricular mass indexed to body surface area in the EMPA-HEART study [115]. However, the REFORM trial, recruiting patients with diabetes and heart failure with reduced ejection fraction (HFrEF), was not able to see any significant remodeling of the left ventricle. However, the study had only 56 patients and most of them had New York Heart Association (NYHA) class I-II, so this was probably an underpowered study. On the contrary, the ATRU-4 study recruited non-diabetic patients with HFrEF NYHA class II-III. After 6 months of follow-up, they were able to demonstrate significant heart remodeling. There was decrease in end-systolic and end-diastolic volumes, decrease in intracellular matrix and the amount of epicardial adipose tissue and decreased arterial stiffness [120]. Remarkable clinical benefits, as well as improved quality of life, were also demonstrated [121]. In a study of 244 patients with 12 weeks of dapagliflozin, there was a reduction in stroke volume and cardiac output, and vascular stiffness, together with mean blood pressure. The systemic changes did not correlate with renal hemodynamic changes. [122]

Single studies reported no change in N-terminal natriuretic propeptide type B (NT-proBNP) in patients with heart failure with reduced ejection fraction [116]. However, the clinical endpoints were improved in acute heart failure [123]. The analysis of participants in the CANVAS program, after 1 year and 6 years, showed a consistent reduction in NT-BNP in patients with canagliflozin versus those without canagliflozin [124].

The EMPEROR-REDUCED study also found decreases in cardiac and renal adverse outcomes in non-diabetic individuals [9]. Diastolic, rather than systolic, function was improved in a small study with empagliflozin [125]. However, another study with the same drug also found improvements in systolic function and reductions in left ventricular mass in patients with HFrEF after 6 months of treatment [126]. 

Empagliflozin reduced extracellular volume in the heart, thus improving the volume of active tissue [127]. Twelve weeks of therapy with dapagliflozin also decreased lung fluid volume [128]. However, myocardial flow reserve was not improved after 13 weeks on empagliflozin [129]. 

The mechanisms underlying these effects have not been completely explored. Decreased energy requirements and the improved mitochondrial metabolism and utilization of ketone bodies are probably the most plausible explanations for this [130,131]. Dapagliflozin was also found to be protective in ischemia reperfusion injury in animal experiment [132]. Luseogliflozin decreases pericardial fat and muscle mass [133]. 

In conclusion, SGLT2 inhibitors improve the functional and structural characteristics of the heart and energy requirements and utilization. Apart from structural changes, all others can be beneficial in the short term and might provide an explanation for short-term cardiovascular benefits.

## 9. Liver Steatosis

Improvements in the concentration of liver enzymes are frequently found in studies. According to recent meta-analyses, gliflozins decrease alanine aminotransferase (ALT) and gamma-glutamyl transferase (GGT) concentrations and decreased the content of liver fat [134]. In a different metanalysis, the results were similar, but aspartate aminotransferase (AST) was also significantly lower [135]. This effect probably does not depend on weight loss only [135]. A small study in India found decreased liver fat on magnetic resonance imaging after 20 weeks on empagliflozin [136], and a similar effect was found in the European population [137]. However, there are no clinical studies that would include liver histology; therefore, changes in liver structure are not directly documented. 

There is some more evidence from experimental data. Empagliflozin was found to decrease liver steatosis similarly to metformin, and change liver transcriptome in a rat model of T2D [138]. Non-alcoholic steatohepatitis (NASH) was also ameliorated, but comparatively with metformin in rodent experiment [139]. A non-obese prediabetic model of hereditary hypertriglyceridemic rats and treatment with empagliflozin were used in another experiment. Hepatokines fibroblast growth factor 21 (FGF21) and fetuin-A decreased in controls and in hypertriglyceridemic rats after treatment. Hepatic glycogen was also significantly decreased [140]. 

As non-alcoholic fatty liver disease (of which NASH is the most severe stage) is a risk factor for cardiovascular outcomes [141], the amelioration of liver function and structure might lead to improved outcomes after SGLT2 inhibition.

## 10. Adverse Effects

SGLT 2 inhibition or absence can also have substantial risks. Mice lacking SGLT2 had better diabetes control than wild-type controls, but they had immensely an increased mortality and rate of infection. This was proportionate to gene loss; heterozygotes had a more favorable outcome than homozygotes. There were also increased urinary losses of magnesium and calcium in these sweet pee mice, and some trend towards an increased excretion of phosphate [142]. The discrepancy between the outcome of these mice and patients with SGLT2 inhibition is striking; however, these mice have very good outcomes unless they were given streptozotocin to make them diabetic. This condition is not comparable to type 2 diabetes. Homozygous mice excreted an enormous amount of urine and glucose. Such a challenge to homeostasis obviously cannot be sustained for a long time. 

In humans, there are very few life-threatening side effects, with dehydration [40], diabetic ketoacidosis (often euglycemic), risk of amputation, genitourinary infections, and fractures being the most prominent. 

SGLT2 increases the concentration of ketone bodies and may lead to euglycemic diabetic ketoacidosis, particularly in patients who lack enough insulin in the system [143]). As this is not the case in non-diabetic patients, no ketoacidosis event has been reported in this population [10]. Apart from the known risks, there is one more, probably underreported, side-effect of ketoacidosis: it might trigger arrhythmias. However, this is probably opposed by the decreased sympathetic activity that results from treatment with gliflozins. A very thorough review on this topic has recently been published [144]. A review of reports to the Food and Drug Administration adverse event-reporting system revealed fewer atrial fibrillation events in patients on SGLT2 inhibitors compared to other antidiabetic drugs [145].

Genitourinary infections are more frequent in patients treated with SGLT2 inhibitors, which is not surprising [38,41,42,146]. There are few studies that did not report this side effect. They are also significantly more frequent in non-diabetic patients [10]. However, these are usually uncomplicated and mostly single episodes [147,148]. 

There is also a concern about bone homeostasis. The increased risk of fractures was found to be significant after more classes of antidiabetic drugs, including SGLT2 inhibitors [53]. However, in most large studies, no increase in fractures was reported. Meta-analyses are not completely conclusive. One of them found a significant increase in fractures after canagliflozin [149]; a more recent study did not find any statistical significance compared to placebo [150].

The higher risk of limb amputations reported in the CANVAS study [5] raised concern. However, most large studies did not find an increased risk and a recent meta-analysis confirmed no increase in amputations in a combined cohort of 63,716 patients [151]. This evidence was consistent across different types of SGLT2 inhibitors, baseline populations and lengths of treatment use. In an analysis including the CANVAS program and CREDENCE trials, history of previous amputation was the strongest predictor of future amputation [152]; thus, the association with treatment was not necessarily causative. On the other hand, impaired recovery from hind limb ischemia was found in experimental diabetic mice treated with canagliflozin [69], so this risk needs ongoing vigilance and more experimental investigation.

Some interesting and non-intuitive findings were reported when gliflozins were combined with training and other lifestyle modifications. Dapagliflozin blunted the improvement in insulin sensitivity induced by endurance exercise [153]. The effect of dietary counselling was also impeded by the same drug [154]. On the other hand, in another study, intensive exercise + dapagliflozin decreased the trunk fat mass [155]. However, the controls received dapagliflozin treatment only, and there was no control with training without treatment. 

The described adverse effects were not numerous and were not severe enough to abolish the beneficial outcomes in the studies cited above.

## 11. Conclusions

Gliflozins decrease the amount of salt and water in the body, which decreases the workload of the heart. The increased availability of ketone bodies and switch of energy consumption from glucose to other sources improves its nutrition. The kidney workload is also decreased as hyperfiltration is abolished and the reabsorption of sodium and glucose is lower, which leads to lowered energy requirements. Both organs benefit from improved blood flow and a better oxygen supply with increased hematocrit. Better mitochondrial function also contributes to the better energy and oxygen utilization in the cells. Decreased sympathetic activity might be a significant contributor to both kidney and cardiac benefits. These effects are rapid and may partly explain the beneficial effects seen during short-term follow-up. 

**Figure 1:**
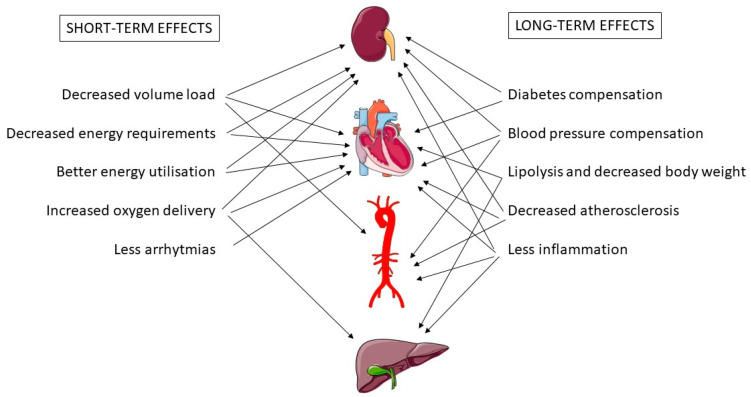
Beneficial effects of SGLT2 inhibitors on kidney, cardiovascular system, and liver.

Improved compensation of diabetes or blood pressure is probably associated with better long-term outcomes after SGLT2 inhibition. In addition, gliflozins might also alleviate the burden of the “thrifty genotype” [156]. Fewer calories and salt are available for the body, even without a change in dietary habits. This effect differs from that of a low-carbohydrate diet because gliflozins help to excrete not only the glucose that was ingested but also glucose that originates from other types of nutrients and gluconeogenesis. The increased lipolysis of white fat has additional benefits for other organs, such as liver. A simplified graphic summary of the beneficial effects of SGLT2 inhibitors is provided in Figure 1.

It is not obvious whether each of the metabolic changes described above contributes to the beneficial effect, and to what extent. Decreased inflammation and atherosclerosis will probably confer even more benefits when long-term follow-up is available. However, the long-term risk of fractures might also increase. As with all new drugs, a thorough and dutiful monitoring of adverse effects is necessary.

SGLT2 inhibitors have attracted a huge amount of interest, which is probably at its peak at present. Their many beneficial effects improve our armamentarium against the burden of civilization diseases. If the side effects are carefully checked and controlled, their benefits are immense. As every functioning human kidney filters glucose, people without diabetes can also profit from the effect of this group of drugs, as was repeatedly shown. 

## Figures and Tables

**Table 1:** Metabolic effects of gliflozins, apart from decreasing glycemia and glycosylated hemoglobin. LDL-cholesterol: low-density lipoprotein cholesterol; HDL-cholesterol: high-density lipoprotein cholesterol.

Parameter	Change
potassium	Less hyperkalemia, hypokalemia not increased [20,21]
magnesium	Increased [22,23]
phosphate	Increased [24]
uric acid	Decreased [25,26]
LDL-cholesterol	Increased, less atherogenic composition [27]
HDL-cholesterol	Increased [27,28]
triglycerides	Decreased [28]
renin	Increased [29]
aldosterone	Increased [29]
parathormone	Increased [22,24]
hematocrit	Increased [30]
